# COST-EFFECTIVENESS OF PERIOPERATIVE IMMUNONUTRITION IN GASTROINTESTINAL ONCOLOGIC SURGERY: A SYSTEMATIC REVIEW

**DOI:** 10.1590/0102-6720201600020014

**Published:** 2016

**Authors:** Audrey Machado dos REIS, Geórgia Brum KABKE, Ana Valéria Gonçalves FRUCHTENICHT, Taiane Dias BARREIRO, Luis Fernando MOREIRA

**Affiliations:** 1 Multidisciplinary Integrated Health in Critical Adult Residency Program; 2 Southern Surgical Oncology Research Group and Post-Graduate Program of Surgery, Faculty of Medicine, Rio Grande do Sul Federal University and Department of Surgery, Hospital de Clínicas de Porto Alegre University Attached Hospital, Porto alegre, RS, Brazil.

**Keywords:** Immunonutrition, Oncologic gastrointestinal surgery, Cost-effectiveness

## Abstract

**Introduction:**

Costs, length of hospital staying and morbidity are frequently and significantly increased as a result of infections and other complications following surgical procedure for gastrointestinal tract cancer. Recently, improving host defence mechanisms have become a target of interest. Immunonutrition aims at improving immunity, most likely providing key nutrients to maintain T-lymphocyte and other host defence.

**Aim:**

To evaluate the immunonutrition in cancer patients who are operated by digestive diseases and assess the cost-effectiveness of this supplementation.

**Methods:**

This study consisted of a systematic review of the literature based on reference analyses retrieved from current databases such as PubMed, Lilacs and SciELO. The search strategy was defined by terms related to immunonutrition [immunonutrition, arginine, omega-3 and nucleotides] in combination with [costs, cost-effective and cost-effectiveness] as well as [gastrointestinal cancer surgery, oesophageal, gastric or pancreatic surgery] in English, Portuguese or Spanish language. For cost analyses, currencies used in the manuscripts were all converted to American dollars (US$) in order to uniform and facilitate comparison. Six prospective randomized studies were included in this review.

**Conclusion:**

The cost-effectiveness was positive in most of studies, demonstrating that this diet can significantly reduce hospital costs in the North hemisphere. However, similar studies needed to be carried to determine such results among us.

## RESUMO

**Introdução:**

Custos, tempo de hospitalização e morbidade estão frequentemente aumentados na presença de infecções e outras complicações decorrentes de procedimentos cirúrgicos para o câncer gastrointestinal. Recentemente, a melhora de mecanismos de defesa do hospedeiro tem se tornado um alvo de interesse. Nutrição adequada está fortemente relacionada com competência imune e redução de infeções. Imunonutrição objetiva a melhora da imunidade, principalmente para manutenção de linfócitos-T e outras defesas.

**Objetivo:**

Avaliar a imunonutrição em pacientes oncológicos que são operados por doenças do aparelho digestivo e avaliar a relação custo-eficácia desta suplementação.

**Métodos:**

Revisão sistemática da literatura baseada nas bases de dados PubMed, Lilacs e SciELO. A busca foi realizada com combinação de descritores em inglês e português relacionados ao tema da revisão: [immunonutrition, arginine, omega-3, nucleotides] combinado com [costs, cost-effective, cost-effectiveness] e [gastrointestinal cancer surgery, oesophageal, gastric or pancreatic surgery]. Para análise de custos, moedas usadas nos artigos foram todas convertidas para dólar americano. Seis estudos randomizados prospectivos foram incluídos nesta revisão.

**Conclusão:**

O custo-benefício foi positivo na maioria dos estudos, sugerindo que este tipo de dieta reduz significativamente os custos hospitalares nos países do hemisfério norte. Contudo, estudos similares de custo-benefício devem ser realizados para definir o real custo-benefício em nosso meio.

## INTRODUCTION

Recently, the relation between infection rates and length of hospital staying (LHS) has been increasing. Surgical procedures involving visceral organs are at a particular high-risk to the patient. Immunity is compromised due to reperfusion and tissue ischemia from stress associated with blood transfusion and haemorrhage^21^.

Moreover, costs, LHS and morbidity are frequently and significantly increased as a result of infections and other complications following gastrointestinal tract (GIT) and head and neck cancer^2^ ^,^ ^16^. Wound infection, abdominal abscess, pneumonia, urinary tract infections are considered postoperative infection complications. Other important complications include: anastomotic leaks, acute renal failure and cardiovascular events^2^. Usually, the policies used to reduce and prevent postoperative complications emphasize on the pathogen eradication as perioperative antibiotic prophylaxis, surgical trauma reduction, intraoperative contamination and improvement in the hospital environment^20^. 

Only recently, improving host defence mechanisms have become a target of interest. Adequate nutrition is strongly linked with immune competence and risk reduction for infections^4^ ^,^ ^14^. Immunonutrition is composed by omega-3 fatty acids (ω-3), arginine and nucleotides aiming to improve immunity, most likely providing key nutrients that maintain T-lymphocyte and other host defence^5^ ^,^ ^13^. 

The aim of this systematic review was to review immunonutrition for oncologic patients who are undergoing surgery for GIT tract and to evaluate the cost-effectiveness of this supplementation.

## METHODS

The study consisted of a systematic review of the literature based on references found in current databases: PubMed, Lilacs and SciELO.

The search strategy was defined by terms related to immunonutrition (immunonutrition, arginine, omega-3 and nucleotides) in combination with headings of hospital costs (costs, cost-effective and cost-effectiveness) as well as oncological patients undergoing GIT surgery (gastrointestinal cancer surgery, oesophageal, gastric or pancreatic surgery).

SciELO and Lilacs did not provide any article. A total of 59 articles were found in a first round. Studies included in the search were those carried out in adults and of English or Portuguese language. No Portuguese articles were found. Reviews, meta-analysis, short or brief communications or those articles that did not have full text available and either methods or subject of the study not detailed stated were also excluded. 

Of the 59 articles, 43 (73%) were duplicated articles and because of duplicity were excluded from analysis. Additionally, four (7%) articles were reviews, three (5%) were meta-analysis, one study was performed exclusively in children (2%), one had confused methods of cost assessment (1.7%), and one (1.7%) have the proposed subject out of our scope, and were all excluded. 

For cost analysis, currencies used in the papers needed to be converted to American dollar in order to uniform and facilitate comparison; for this purpose was considered the first day of the month and the year that the paper was submitted or published as stated accordingly. Also all currencies have cost values updated by November 1^st^ 2014 as calculated by assessing a European Central Bank website for the purpose of currency updating^6^. Two papers had Deutsche Mark as currency ^17^ ^,^ ^18^, one had Chinese Yuan Renminbi^23^ and the remaining ones used the Euro. In only one late article, the publishing date did not allow currency conversion for the whole period^18^. More details are shown in Table 1.

**TABLE 1:** Currency conversion details

Author, year	Currency	Article date	Euro Exchange Rate
Senkal M et al., 1999	DM	December 1999	€1 = 1.9558 DM €1 = 1.0091 US$
Gianotti L et al., 2000	EURO	September 2000	€1 = 0.8902 US$
Kłek S et al., 2005	EURO	April 2005	€1 = 1.2959 US$
Braga M et al., 2005	EURO	July 2004	€1 = 1.2168 US$
Zhu M et al., 2012	RMB	January 2012	€1 = 8.1588 RMB €1 = 1.2939 US$

DM=Deutsche Mark; EURO=European Community currency; RMB=Chinese Yuan Renminbi

## RESULTS

Six prospective randomized studies were included in this review and these articles are summarized in Table 2. The GIT cancer analyzed were: GIT cancer in general (n=3), colon or rectal cancer (n=1), upper GIT cancer (n=1) and gastric cancer (n=1). In all articles ω-3 supplement use was described. Arginine and RNA were used as supplements in four studies and glutamine supplementation was used in only one study. GIT supplements were given by parenteral supplementation (n=2), oral (n=3; preoperative cases only) or enteral feeding (n=4) in the studies revised. Table 3 depicts more detailed characteristics of the study groups such as sample size, mean age and gender ratio.

**TABLE 2:** Article descriptions

Author, year, Country	Journal, Study	Sample, cancer type	Diet	Diet administration	Supplementation via
Senkal M et al., 1997. Germany	Crit Care Med Prospective, randomized, double-blind study and a retrospective cost-comparison analysis.	154 patients Upper GIT cancer	Treatment group: diet supplemented with arginine, omega 3 and RNA Control group: isonitrogenous and isocaloric liquid diet.	Starting in the 1st POD.	Enteral Nutrition
Senkal M et al., 1999. Germany	Arch Surg. Prospective, randomized, double-bind study.	154 patients GIT cancer	Treatment group: diet supplemented with arginine, omega 3 and RNA Control group: isocaloric liquid diet.	5 days prior to surgery to 5th POD.	Preoperative: oral Postoperative: enteral
Gianotti L et al., 2000. Italy	SHOCK Prospective, randomized, double-bind study.	206 patients GIT cancer	Treatment group: diet supplemented with arginine, omega 3 and RNA Control group: isonitrogenous and isocaloric liquid diet.	7 days prior to surgery to 7th POD.	Preoperative: oral Postoperative: enteral
Kłek S et al., 2005 Cracow	Acta Chir Belg Prospective, randomized study	90 patients Stomach cancer	Group Control: standard diet; Group B: diet supplemented with glutamine Group C: diet supplemented with omega-3.	2nd to 9th POD or until enteral diet covered at least 60% of protein and energy requirements.	Parenteral Nutrition
Braga M et al., 2005. Italy	Nutrition Prospective, randomized study and a retrospective cost-comparison analysis	305 well-nourished patients GIT cancer	Diet supplemented with arginine, RNA and omega-3.	Preoperative group - supplementation 5d prior surgery Perioperative group - same as preoperative treatment plus specialized diet up to 7th POD	Preoperative: oral supplementation Postoperative: enteral supplementation
Zhu M et al., 2012. China	Chin Med J. Prospective, randomized, double-blind study	57 Elderly patients body BMI of 18.5-25.0 kg/m² Colon or rectal cancer	Treatment group: 0,2 g/Kg fish oil and 1,0 g/Kg soybean oil Control group: 1,2 g/Kg soybean oil	Both groups had diet from 1st to 8th POD	Parenteral Nutrition

POD = Postoperative day

**TABLE 3:** Detailed study group characteristics

Author, year	Sample Size		Age (years)		M: F ratio	
	Control Group (N=417)	Treat. Group (N=438)	Control Group (SD)	Treat. Group (SD)	Control Group	Treat. Group
Braga M et al., 2005	102	102*	68.1 (11.7)	69.4 (10.1)*	56:46	50:52*
Zhu M et al., 2012	28	29	70.8 (6.4)	69.8 (10.5)	17:11	16:13
Senkal M et al., 1999	76	78	67.0 (9.0)	64.0 (11.0)	48:30	52:24
Gianotti L et al., 2000	104	102	61.1 (9.5)	60.8 (11.5)	42:62	39:63
Senkal M et al., 1997	77	77	66.3 (1.8)	65.1 (1.5)	NA NA	
Kłek S et al., 2005	30	31 (Group B) 29 (Group C)	Total Sample: 61.9		Total Sample: 51:39	

Group B=glutamine supplemented group; Group C=omega-3 supplemented group; Treat.=treatment; M F=male female; *=preoperative treat. only; SD=standard deviation

Nutritional status is described in Table 4. Albumin, pre-albumin and weight-loss were chosen in three studies to define nutritional status. Body mass index (BMI) and Nutritional Risk Index both were observed in two articles to define nutritional status. One study selected just well nourished patients^10^. The other studies have not restricted nutritional status. Zhu *et al*. ^23^ selected only elderlies (65 to 85 years old) that had 18.5-25.0 kg/m² BMI. Klek *et al*.^11^ classified subjects in "well-nourished" and "minor grade malnutrition", and then equally distributed sample into the groups. 

**TABLE 4:** Patient nutritional status

Author, year	Sample Nutritional Status	Albumin (g/ml)		Pre-albumin (mg/dl)		Weight Loss (%)		BMI		Nutrition Risk Index	
		Control Group (SD)	Treat. Group (SD)	Control Group (SD)	Treat. Group (SD)	Control Group (SD)	Treat. Group (SD)	Control Group (SD)	Treat. Group (SD)	Control Group (SD)	Treat. Group (SD)
Braga M et al., 2005	Well-nourished patients	40 (6.5)	40 (5.6)*	0.2 (0.07)	0.3 (0.08)*	2 (2.7)	2 (2.6)*	NA		NA	
Zhu M et al., 2012	Different nutritional status	NA		NA		NA		23.2 (3.6)	22.9 (3.1)	NA	
Kłek S et al., 2005	Different nutritional status	NA		NA		NA		NA		NA	
Senkal M et al., 1999	Different nutritional status	NA		NA		NA		23.2 (3.6)	22.9 (3.1)	91 (15)	97 (12)
Gianotti L et al., 2000	Different nutritional status	39 (11)	38 (10)	NA		5 (4.1)	6 (4.2)	NA		NA	
Senkal M et al., 1997	Different nutritional status	NA		NA		NA		NA		98 (1.7)	99 (1.9)

*=Preoperative group only; Treat.=treatment; BMI=body mass index; NA=not available; SD=standard deviation

### Reduction of complications

Overall, the studies found a reduction of complications in all groups receiving some kind of immunonutrient. Five of the manuscripts demonstrated a statistically significant difference on complications^3^ ^,^ ^7^ ^,^ ^17^ ^,^ ^18^ ^,^ ^23^. On the other hand, considering Intensive Care Unit admissions, two studies did not present any protection by supplementation^3^ ^,^ ^7^.

Braga *et al*.^2^ supplementing with arginine, RNA and ω-3 showed a significant decrease in the number of patients who developed postoperative infections in both treatment groups that received immune-enhancing diets as compared to controls. Though, complications were not isolated analysed by groups; they were classified as major (infections) or minor (non-infections) complications. Both, major and minor complications were reduced in the groups with patients receiving immunonutrition. There were 42 major complication events (18 in the control group, 10 and 14, in the preoperative and perioperative groups, respectively) and 157 minor complications (67 in controls, 44 in the preoperative and 46 in the perioperative groups). Eight patients were transferred to intensive care (four in the perioperative group, three in the controls and only one in the preoperative). 

Gianotti *et al*.^7^ supplemented arginine, RNA and ω-3 in GIT cancer patients as well. They found that the number of complications was significant lower (except for peritonitis) in the treatment group; where less anastomotic leak and pneumonia was observed. Two and three treated and non-treated patients were sent to intensive care, respectively. The same immunonutrients were analyzed in another randomised study, including 18% of the patients with GIT cancer showing postoperative complications. After the 3^th^ postoperative day, the number of patients who developed complications was significantly lower in the treatment group than in controls. Moreover, the number of patients who had late complications was suggestively lower in the treatment group when compared to controls^18^.

Upper GIT cancer patients were observed in a study using arginine, RNA and ω-3 combined. A total of five deaths occurred, three in the group receiving the studied diet and two controls receiving standard formula. The causes of death in the treatment group were systemic inflammatory response syndrome (SIRS; n=2) and myocardial infarction (n=1). Two eligible patients who received standard diet died because of cardiopulmonary complications. The number of patients with complications clearly decreases as for postoperative day 4 under immunonutrition, considering the number of patients with complications in control group remained almost unchanged till postoperative day seven. Among 77 eligible patients, 17 and 24 in the supplemented diet and non-supplemented groups experienced postoperative complications, respectively. Also, late complications were much more observed in controls than in treatment group (5 vs. 13 cases; p<0.05). However, prevalence of complicating events was not significantly decreased in the supplemented diet group as compared to controls; 30 vs. 32 cases, respectively^17^.

Elderlies with colon or rectal cancer were analysed by Zhu *et al*.^23^. Eight patients in the control group (five respiratory tract infections, one urological and two wound infection) as compared to four (three respiratory tract infections and one wound infection) in the treatment group that received fish oil (p>0.05) had postoperative complications. Besides that, fish oil significantly reduced the incidence of SIRS (p<0.05). Omega-3 fatty acids were also observed in the study of Kłek *et al*.^11^ and pneumonia was more frequently observed in the control group, but no significant differences were seen between immunomodulation and control groups.

Two deaths occurred in a treatment group because of SIRS^17^ while another study observed a prevention effect^23^. However, in that study deaths were noted; ω-3 was offered combined with arginine, while in the second study^23^ was found protection to SIRS when isolated omega-3 fatty acids was offered. Studies done with critical patients pointed that the use of arginine in septic patients was associated with higher mortality rates^10^ ^,^ ^12^, suggesting that arginine, by increasing pro-inflammatory cytokines and nitric oxide, increases inflammatory response due to toxic effects; and these effects seemed to be greater in patients with severe infection, sepsis or SIRS^19^. Moreover, omega-3 fatty acids significantly reduced the incidence of SIRS in another studies as well^9^ ^,^ ^15^ ^,^ ^22^. 

### Length of hospital staying

All studies that analyzed LHS found that supplemented patients had a shorter hospital admission. Zhu et al.^23^ showed that mean (SD) LHS in treatment group significantly decreased as compared to control group; 12(4) days and 15(6) days, respectively (p*<*0.05). Senkal *et al*.^18^ and Kłek *et al*.^11^ have not found significantly differences between control and treated groups. 

Patients receiving ω-3 supplementation resulted in shorter LHS with a mean (SD) days in hospital - 14(4) days - when compared to those receiving glutamine supplementation - 14(8) days - and to controls - 16(4) days. The range was also greater for controls (9 to 45 days) when compared to ω-3 (9 to 42 days) and glutamine (8 to 41 days) supplementation^11^. Senkal et al. ^14^ supplementing patients with ω-3, arginine and RNA diet found that these patients had a mean (SD) LHS of 5.1 (1.2) days in intensive care *vs*. 6.8 (1.4) days for controls. Total LHS (SD) was 27 (2.3) days in the supplemented diet group vs*.* 30.6 (3.1) days for controls. The same immunonutrient combination was given by Braga *et al.* *^2^* who separately analyzed LHS by cancer type in patients without complications. Mean (SD) LHS values for patients who underwent gastroesophageal resection were 10.7 (3.9) days in the control group and 9.9 (4.2) in the preoperative group. Mean (SD) LHS values for patients who underwent pancreatic resection were 13.8 (6.1) days in the standard diet group and 12.7 (5.8) days in the preoperative group. The mean (SD) LHS values for patients who underwent colorectal resection were 8.8 (4.0) days in the control group and 8.4 (3.7) days in the preoperatively treated group. These studies, clearly demonstrated the benefit of supplementation over no supplementation concerning LHS.

### Cost-effectiveness

As expected, the supplemented diet costs were higher than standard diet for all studies. Overall supplemented diet costs ranged from US$ 14 to US$ 101 per-patient while standard diet costs ranged from US$ 22 per-patient to US$ 348 per-patient. These costs are shown in Table 5.

**TABLE 5:** Supplemented diet costs

Author, year;	Nutrition Cost	
	Control Group	Treat. Group
Braga M et al., 2005	4,146 US$ 41 US$ per patient	17,922 US$ 176 US$ per patient
Zhu M et al., 2012	407 ±70 US$	638 ±49 (<0.01) US$
Senkal M et al., 1999	25 US$ per patient	179 US$ per patient
Gianotti L et al., 2000	91 US$ per patient (intent-to-treat analysis) 101 US$ per patient (core analysis)	309 US$ per patient (intent-to-treat analysis) 348 US$ per patient (core analysis)
Kłek S et al., 2005	Not available	8,668 US$ (<0.5) 299 US$ per patient *

Omega 3 supplemented group only*

Braga *et al*.^2^ showed cost of each complication based on LHS and resources used for major complications, where the largest mean cost was for sepsis (US$ 16,669) occurring in three patients who had the most expensive resources used (US$ 15,173). Abdominal abscess and anastomotic leak had the largest mean spending due to prolonged LHS. For minor complications, wound dehiscence that occurred in seven patients had the most expensive mean (US$ 7,740), also mainly due to prolonged LHS. Intestinal obstruction (n=2) with the largest mean cost (US$ 3,340) and a single instance of pulmonary embolism with expenses of US$ 1,940 were higher because of used resources. No significant difference was found after comparing the mean cost of each complication between the three treatment groups (perioperative or preoperative supplementation vs. control). 

Senkal *et al.* ^18^ analyzing complications found that the most expensive early complication was pneumonia in supplemented group (US$ 6,008), occurring in only one case, while late complications showed the largest expenses for intensive care admissions, pneumonia and sepsis (US$ 21,499) in the supplemented (n=6) group, and pneumonia, anastomotic leak and pancreatitis (US$ 55,226) in controls (n=17); which were higher in both, number of cases and overall expenses. Costs for treating postoperative complications were US$ 497 and US$ 1,387 per-patient in the group receiving immunonutrition vs*.* controls, respectively. Gianotti *et al.* ^7^ had also found that immunonutrition reduces costs of complications. In their series, mean total cost per complication was US$ 3,874 for the treatment group as compared to US$ 6,385 in controls. Costs by intent-to-treat analysis were also significantly lower in the treatment group (US$ 2,660) against US$ 6,431 for controls (core analysis; p=0.05). Moreover, total costs and costs to treat postoperative complications by intent-to-treat analysis accounted for US$ 69,735 vs. US$ 217,104 and US$ 37,251 *vs.* US$ 205,786, respectively for the immunonutrition group vs. controls. The most expensive treatment in the supplemented group was peritonitis (US$ 17,978) and by the intent-to-treat analysis (n=1) was anastomotic leak (n=5), mean (SD) cost of US$ 5,390 (2,591). Anastomotic leak (n=10) was also the most expensive treatment of complications in the control group (US$ 14,038) by both analysis. 

Worth of note was that a rough analysis based on LHS demonstrated that immunonutrition would not be cost-effective, since overall costs reached US$10,885 with standard diet (controls), US$ 11,075 with glutamine and US$ 13,672 with ω-3 supplementation. However, authors did not evaluate, complication costs, thus compromising the usefulness of immunonutrition, indeed^11^. 

Cost-effectiveness was positive in the study by Gianotti *et al*.^7^, who found immunonutrition overcompensating costs with postoperative infection resulting in a significant net saving for infection complication treatment of US$ 1,186 and US$1,484 by intent-to-treat and by core analysis per complication-free patient, respectively. Total costs in a cost-effectiveness analysis showed a saving of US$ 2,124 by intent-to-treat analysis and of US$ 2,416 by core analysis. Overall costs were US$ 8,498 in the treatment group vs*.* US$ 12,060 in the control group, i. e., a saving of US$ 3,562 favouring immunonutrition. 

The most recent study^11^ on cost-effectiveness did not find a statistically significant difference on total medical care costs (nutritional plus non-nutritional) between supplemented and non-supplemented cases (US$ 6,030 vs. US$ 6,021). On the other hand, although Senkal *et al*.^18^ did not find a statistically significant difference in the mean treatment costs per patient, they did demonstrate a 32% saving when overall complication costs were analyzed. Later (1999), however, those authors showed a net saving of US$ 1,439 per-patient with a cost-effectiveness also favouring immunonutrition demonstrating that total costs in the supplemented group were almost a third (US$ 75,857) of the costs of the controls (US$ 206,099). 

Additionally, Braga *et al*.^2^ also reported a net saving of total costs of US$ 176,780 for those cases receiving immunonutrition who had a favourable postoperative course. Cost-effectiveness per patient was US$ 2,280 in the preoperative group and US$ 3,799 in the conventional group, i. e., a saving of US$ 1,519 (p=0.04); and when the analysis was limited to cases complicated by infection the cost-effectiveness was significantly greater in preoperative supplemented diet group as compared to the standard diet group (US$ 2,990 vs*.* US$ 956; p=0.01). This trend however, was not observed with non-infection complicated cases, where no statistically significant difference was found. 

A randomized clinical trial not included in this review, concluded that for malnourished patients, where only a single study included only well-nourished patients, the use of preoperative nutritional approach seemed to be more clinically beneficial than only postoperatively^1^, since sole preoperative immunonutrition can be clinically and economically enough. This may explained because malnourished patients have increased energy and nitrogen needs and decreased immune response, and therefore, prolonged administration of immunonutrients can be indicated^3^. 

The present systematic review showed that there are great advantages in the use of a diet with immunonutrients, even though its cost-effectiveness is still seeing with some scepticism and under scrutiny. Although this review included a small number of studies, that may difficult wider interpretations, it assessed a number of manuscripts restricted by the target subject with a quite good number of cases (n=417) and controls (n=438); in which results consistently demonstrated, for instance, if immunonutrition is attempted a decrease on LHS and complication rates lead to a significant reduction in the overall patient expenses should be clearly expected. 

For economic analysis, some limitations may have partially influenced outcomes, since costs in older articles, may have been economically underestimated and in some cases, articles have not precisely informed the date of cost analysis. Furthermore, economic parameters used in the analysis may differ from institution to institution in each country based on each hospital billing system and reimbursement rates^3^ and therefore, more studies on cost-effectiveness needed to be carried out. In Brazil, as far we know, there is no actual data about cost-effectiveness of immunonutrition^8^, a lack we intend to fulfil with an on-going study on immunonutrittion in patients with upper GIT tumours. 

## CONCLUSION

Immunonutrition reduces complications and LHS, perhaps with the exception of intensive care admissions and deaths. The cost-effectiveness was positive in all studies and savings were significant in most of studies, showing that this approach can be worth. Though, external validation of the results cannot be secured to any region due to differences between the health care, billing system and currencies from country to country.
